# Hand Amputations

**Published:** 2018-09-28

**Authors:** Matthew P. Fahrenkopf, Nicholas S. Adams, John P. Kelpin, Viet H. Do

**Affiliations:** ^a^Michigan State University College of Human Medicine, Grand Rapids; ^b^Spectrum Health/Michigan State University Plastic and Reconstructive Surgery Residency, Grand Rapids; ^c^Orthopaedic Associates of Michigan, Grand Rapids

**Keywords:** upper extremity, hand, amputation, prosthetics, infection

## DESCRIPTION

**Figure 1:**
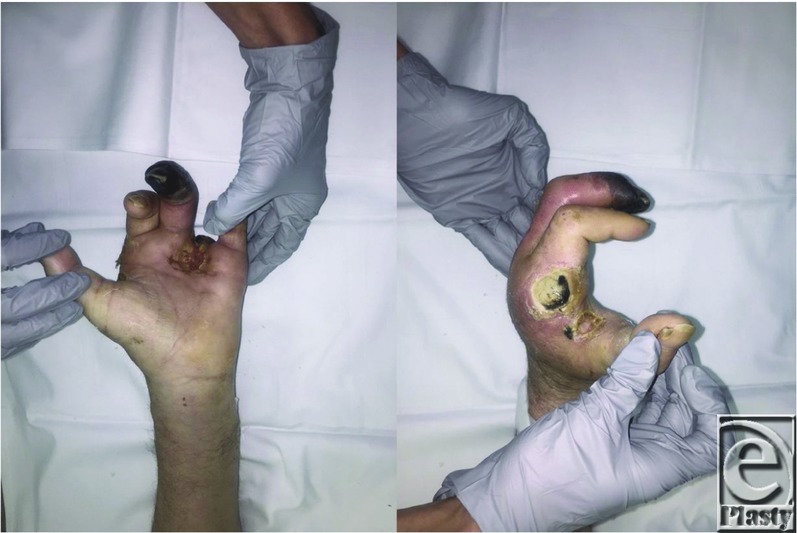
Nonhealing left ring finger revision amputation and newly developed hand wounds.

A 60-year-old man with a medical history of renal disease, diabetes, and peripheral vascular disease presented to the emergency department for left ring finger gangrene and cellulitis (Fig 1). Failure of conservative management resulted in a ring finger revision amputation. With his underlying medical comorbidities, the patient's wound never healed and developed multiple nonhealing wounds of the left hand, necessitating an amputation.

## QUESTIONS

1. What is the incidence of upper extremity amputation?
2. What are the possible locations for upper extremity amputation?
3. How does amputation location affect prosthesis fit and use?
4. How do you perform a forearm amputation?

## DISCUSSION

There are an estimated 1.7 million people in the United States living with limb loss, and 50,000 to 100,000 new amputations occur per year.^1^^,^^2^ The location of these amputations is disproportional, with lower extremity amputations occurring at a rate of 4-5:1 compared with upper extremity amputations.^3^ Seventy percent of patients with upper limb loss undergo amputation below the elbow, with 10% of those occurring at hand or wrist.^1^^-^3 The major causes of these upper extremity amputations include trauma, malignancy, vascular disease, congenital deformities, and infection.

Upper extremity amputations are typically divided into 2 groups: (1) below-the-elbow and (2) above-the-elbow amputations. Below-the-elbow procedures encompass transcarpal amputations, wrist disarticulations, and forearm amputations. Above-the-elbow procedures encompass transhumeral amputations, shoulder disarticulations, and forequarter amputations. Where an amputation is performed depends on the indication (ie, trauma, malignancy, congenital, etc), soft tissue viability, prosthesis requirement, and the degree of native motion the physician would like to preserve. Wrist disarticulation offers the benefit of preservation of the distal radioulnar joint and length, with the drawback of a bulbous stump more difficult to accommodate prostheses.^4^ Amputations through the distal, mid, and proximal forearm maintain approximately 90%, 70%, and 30% of pronation and supination, respectively.^5^ If at least 4 cm of proximal ulna is maintained, elbow flexion will remain intact.^6^

Amputations impact not only patients’ emotional quality of life but also their functionality in everyday life. Therefore, the surgeon must be aware of the outcomes associated with each level of amputation. Preservation of length in the upper extremity is paramount, as a longer lever-arm allows for greater torque generation.^4^ This torque can be utilized to power prosthetics with little force on the underlying soft tissue. These prostheses help restore form and function of the lost limb and may help ease patients’ social anxiety. Forearm prostheses are the best tolerated, followed by ones for transhumeral amputations and shoulder disarticulations. Rejection rates of upper extremity prostheses approach 38% in proximal amputations.^5^ Behrend et al^7^ identified increasing prosthetic weight, phantom pain, socket discomfort, lack of sensory feedback, and lack of functionality as risk factors for prosthesis rejection. These all increase as one moves proximally on the upper extremity. A study examining the outcomes of upper extremity amputations found that individuals with below-the-elbow amputations performed 2-handed activities more easily than did persons with above-the-elbow amputations; however, there was no significant difference between the 2 groups in performing activities of daily living.^8^

**Figure 2:**
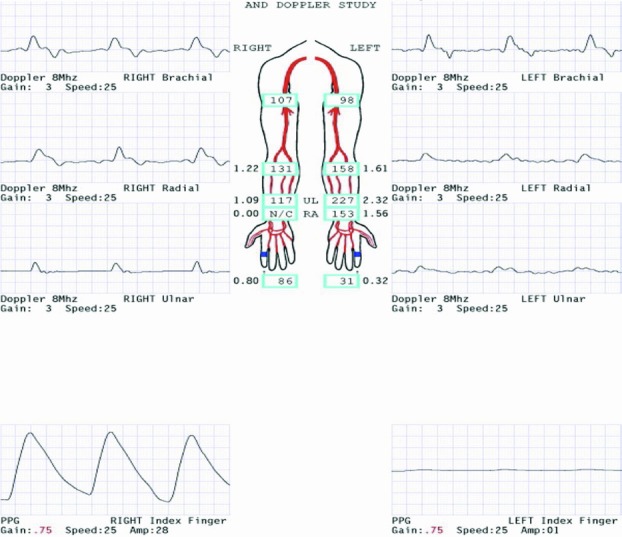

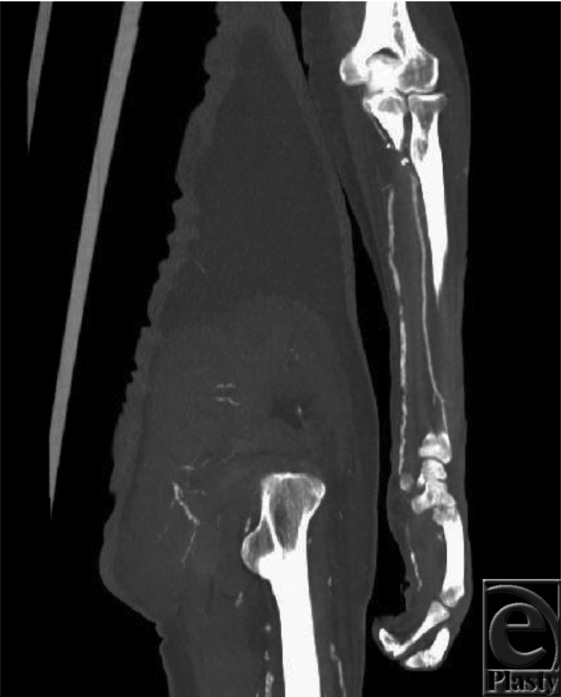
(a) Doppler ultrasonography report showing poor left hand perfusion. (b) Computed tomography angiography of left upper extremity demonstrating calcified arteries and diminished flow around the level of the wrist.

As proposed by Ernest Burgess, amputation is not a failure but rather the first step in a reconstructive and rehabilitation process. After a thorough clinical examination, adjunctive tests can be utilized to determine the amputation level. Examples include arteriography, ultrasonography, transcutaneous oxygen levels, magnetic resonance imaging, and computed tomography. Our patient underwent Doppler ultrasonography and computed tomography angiography to help determine the best level for amputation, which was found to be the mid-distal forearm (Figs 2*a* and 2*b*).

**Figure 3:**
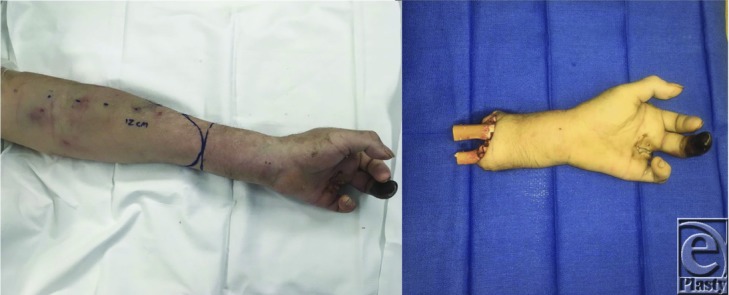
Planned fish mouth incision at the mid-distal forearm (left) and distal amputated segment (right).

**Figure 4:**
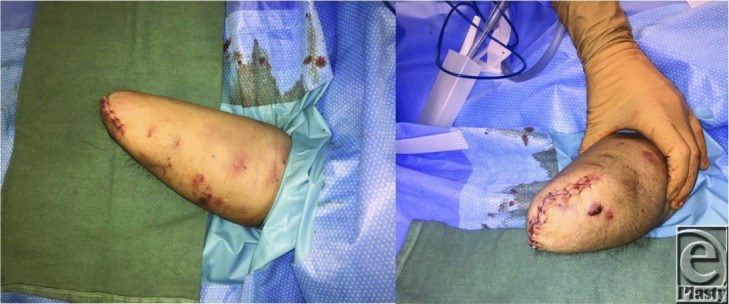
Amputation stump after mid-distal forearm amputation.

Once the amputation level has been decided, a fish mouth incision is designed over the extremity (Fig 3). Healthy soft tissue flaps are elevated proximally. The flexor and extensor tendons are identified and sutured together as 2 separate groups. Vital neurovascular structures are then identified and suture ligated, ensuring they will retract or be buried under robust soft tissue. Osteotomies are made, and remaining sharp edges are trimmed. The flexor and extensor groups are then sutured together over the distal stump. This places tension on forearm musculature, improving the proprioception and the function of the remaining stump. Finally, the soft tissue flaps are reapproximated to close the skin (Fig 4). Ideally, a prosthesis should be fitted within 30 days postoperatively.^5^^,^^6^

Upper extremity injuries can produce profound effects on patients’ lives. Although perceived as morbid, amputations are more often than not lifesaving surgical procedures. When performed properly, amputations can salvage limbs, preserve maximal residual function, and decrease long-term complications such as neuroma, phantom limb pain, and wound complications.
